# Using a Further Planning MRI after Neoadjuvant Androgen Deprivation Therapy Significantly Reduces the Radiation Exposure of Organs at Risk in External Beam Radiotherapy of Prostate Cancer

**DOI:** 10.3390/jcm12020574

**Published:** 2023-01-10

**Authors:** Roland Merten, Mirko Fischer, Hans Christiansen, Susanne Hellms, Christoph Alexander Joachim von Klot, Nele Henrike Thomas, Anne Caroline Knöchelmann

**Affiliations:** 1 Clinic for Radiotherapy, Hannover Medical School, 30625 Hannover, Germany; 2 Institute for Radiology, Hannover Medical School, 30625 Hannover, Germany; 3 Clinic for Urology, Hannover Medical School, 30625 Hannover, Germany; 4 Institute for Biostatistics, Hannover Medical School, 30625 Hannover, Germany

**Keywords:** prostate cancer, radiotherapy, MRI-based treatment planning, androgen deprivation therapy, GI-toxicity

## Abstract

Radiotherapy for prostate cancer is often preceded by neoadjuvant androgen deprivation therapy (ADT), which leads to a reduction in the size of the prostate. This study examines whether it is relevant for treatment planning to acquire a second planning magnetic resonance imaging (MRI) after ADT (=MRI 2) or whether it can be planned without disadvantage based on an MRI acquired before starting ADT (=MRI 1). The imaging data for the radiotherapy treatment planning of 17 patients with prostate cancer who received two planning MRIs (before and after neoadjuvant ADT) were analyzed as follows: detailed comparable radiation plans were created separately, each based on the planning CT scan and either MRI 1 or MRI 2. After ADT for an average of 17.2 weeks, the prostate was reduced in size by an average of 24%. By using MRI 2 for treatment planning, the V60Gy of the rectum could be significantly relieved by an average of 15% with the same coverage of the target volume, and the V70Gy by as much as 33% (compared to using MRI 1 alone). Using a second MRI for treatment planning after neoadjuvant ADT in prostate cancer leads to a significant relief for the organs at risk, especially in the high dose range, with the same irradiation of the target volume, and should therefore be carried out regularly. Waiting for the prostate to shrink after a few months of ADT contributes to relief for the organs at risk and to lowering the toxicity. However, the use of reduced target volumes requires an image-guided application, and the oncological outcome needs to be verified in further studies.

## 1. Introduction

Prostate cancer remains one of the most common yearly cancer diagnoses in many parts of the world [1]. According to the guidelines, radiotherapy in addition to surgery is a curative option in patients with localized disease without distant metastases [2,3]. In patients with high or (at least unfavorable) intermediate risk, radiotherapy should be accompanied by androgen deprivation therapy (ADT) for 6 months in intermediate [4] or at least 18 to 24 months in high-risk patients [5,6]. Therefore, ADT is often started not less than 2–3 months before the initiation of radiotherapy as a neoadjuvant treatment [7]. In addition to the prognostic value of the PSA response to neoadjuvant treatment [7], another aim of such a neoadjuvant approach is to decrease the prostate volume before the start of irradiation with the subsequent possibility of also reducing the target volume of irradiation and thereby lowering the doses to the organs at risk and the risk for clinically relevant toxicity [8].

Because of the superior soft tissue contrast, the implementation of magnetic resonance imaging (MRI) in radiotherapy treatment planning for prostate cancer beyond the essential planning computed tomography (CT) is standard [9,10], and different MR-CT image registration methods are used for this purpose [11].

In clinical practice, at the first radio-oncological presentation, patients often present with an MRI of the prostate/pelvis, which was performed as part of the initial diagnostics. In the case of neoadjuvant androgen deprivation therapy, radiotherapy is usually planned after 2–3 months of ADT (see above) based on an actual planning CT. In many cases, an actual further MRI is also performed at this time. The reason for this is the assumption of decreased prostate volume after neoadjuvant ADT so that an actual MRI is necessary for MR-CT image registration and treatment planning. However, such an approach only makes sense if the target volume and continuing the doses to the organs at risk can truly be significantly decreased using actual imaging, secondarily leading to a lower risk of radiation-induced side effects. If this was not the case, the initial MRI could be used for treatment planning. This question has, to our knowledge, not yet been finally answered in the literature, although the performance of a second MRI is often used routinely in clinical practice.

Therefore, the aim of the present study was to examine whether planning with an actual MRI after neoadjuvant ADT (MRI 2) in definitive radiotherapy for prostate cancer truly leads to optimization of the radiation plan compared to the usage of the initial MRI (MRI 1). Because of the limited availability of MRIs, the use of the old MRI (MRI 1) for radiation planning is common in clinical practice. The present study investigated whether the acquisition of a second MRI may be dispensable or is truly indispensable.

## 2. Materials and Methods

### 2.1. Patients

The imaging data for radiotherapy treatment planning of 17 patients with localized prostate cancer who had received an MRI at initial diagnosis (MRI 1) and CT (Somatom, 16 slices, Siemens, Berlin, Germany) and a further MRI (MRI 2) after neoadjuvant ADT were analyzed. Selected from patients in our outpatient unit in 2020–2021 were all 17 who received neoadjuvant ADT and two MRIs. No further medical selection of patients took place. Detailed characteristics of the patients are given in Table 1.

### 2.2. Radiation Treatment Plans

For all patients, prostate and seminal vesicles were contoured in compliance with the ESTRO Consensus Guideline for Target Volume Delineation [14] and with the anatomical definitions from known publications [9,15,16]. To keep interobserver variability low [17], the prostate was contoured by two independent radiotherapists and radiologists on MRI 1 and MRI 2 at the same level as fusion with the planning CT. To determine the interobserver variability, the volumes were subtracted from each other, and the difference was related to the volume. The Clinical Target Volume for prostate and seminal vesicles (CTV1) was calculated from the delineated organs plus a margin of 5 mm in all directions, and the corresponding planning target volume (PTV1) was calculated from the CTV plus another margin of 5 mm in all directions. The CTV2 of the boost target volume was calculated from the prostate plus a margin of 3 mm, and the corresponding PTV2 was calculated from the CTV2 with a further margin of 3 mm. For PTV1, a VMAT-technology treatment plan was calculated up to a prescribed dose of 60 Gy. For the boost (PTV2), a VMAT plan with a serial dose of 14 Gy was calculated as a sequential boost. The plans were then summed to a total cumulative dose of 74 Gy with a 2 Gy single dose each. All plans were calculated separately based on MRI 1 or MRI 2 after fusion with the planning CT. To make all plans fully comparable, irradiation of the target volume according to ICRU 83 [18] was needed: D_2%_ of the PTV was not allowed to exceed 107% of the prescribed dose, and D_98%_ in the PTV was not allowed to be less than 95% of the prescribed dose, resulting in a heterogeneity index between 1.06 and 1.12 (calculated according to Liu et al. [19]). The mean heterogeneity index in the collective of this study was 1.08. The conformity index of the plans was calculated according to Feuvret et al. [20] Values between 0.77 and 0.96 were allowed for the conformity index so that all plans were also comparable in conformity. The mean value of the conformity index was 0.88 in this study. Thus, the calculated plans based on MRI 1, MRI 2, and CT were standardized and comparable with respect to conformity and heterogeneity. Treatment planning and image fusion was performed using Monaco (Elektra, Stockholm, Sweden). In the Pareto optimization to spare the organs at risk, the rectum was given priority over the urinary bladder because toxicity at the rectum is the largest problem in routine clinical practice [21]. The radiation exposure of the organs at risk was taken from the cumulative plans for the total cumulative dose of 74 Gy, and the following parameters were assessed as relevant: for the rectum and urinary bladder, the mean dose and the percentage of the organ volume higher exposed than 50 Gy V_50Gy_, V_65Gy_, and V_70Gy_; for the rectum, the V_60Gy_ was additionally compared.

All irradiation schedules were fictitious. The treatment of the patients was completely independent of this study and had already been completed at the time of the study. Only imaging was used.

### 2.3. Statistics

Repeated-measures ANOVA with a two-sided α of 0.05 was applied to compare the different plans for each organ or PTV. If statistically significant, pairwise *t* tests for dependent samples were subsequently performed, again with a two-sided α of 0.05. A *t* test for dependent samples (α = 0.05) was also used to compare the prostate volume before and after ADT. The *p* values < 0.05 for the repeated-measures ANOVA and the (pairwise) *t* tests were considered significant. Due to the retrospective nature of the data, no multiplicity concerns arose.

To measure interobserver variability, the CTV of observer one was subtracted from the CTV of observer two for both MRI 1 and MRI 2. The difference was compared separately with and without regard to the sign so that positive and negative differences could not cancel each other out when the average was calculated.

## 3. Results

**Figure 1:**
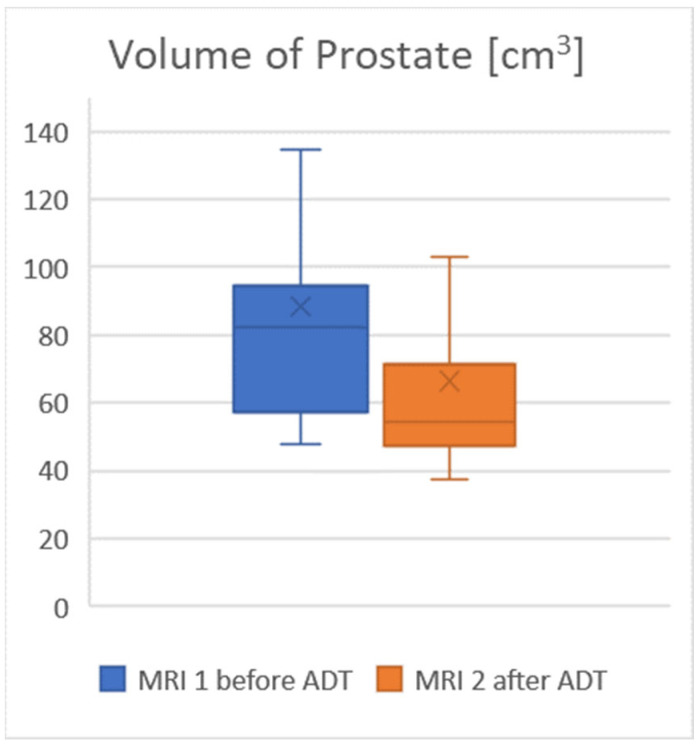
The volume of the prostate decreased significantly under ADT.

The average prostate volume was 88 cm^3^ on MRI 1 (Observer 1: 88 cm^3^, range 48–226 cm^3^. Observer 2: 82 cm^3^, range 44–197 cm^3^) and 66.5 cm^3^ on MRI 2 (Observer 1: 66 cm^3^, range 38–182 cm^3^, Observer 2: 61 cm^3^, range 37–158 cm^3^), which was considered to be a significant change, with a *p* value < 0.05. The average interobserver difference was 6.8%. After an average duration of ADT of 17.2 weeks, the prostate decreased in size by an average of 24%. For further details, see Table 2 and the visualization in Figure 1.

PTV1 (prostate and seminal vesicles) decreased significantly, on average, by 14% after ADT from 295 cm^3^ on MRI 1 to 255 cm^3^ on MRI 2. The boost PTV2 decreased significantly, on average, by 15% after ADT from 148 cm^3^ on MRI 1 to 126 cm^3^ on MRI 2. For further details, see Table 3.

**Figure 2:**
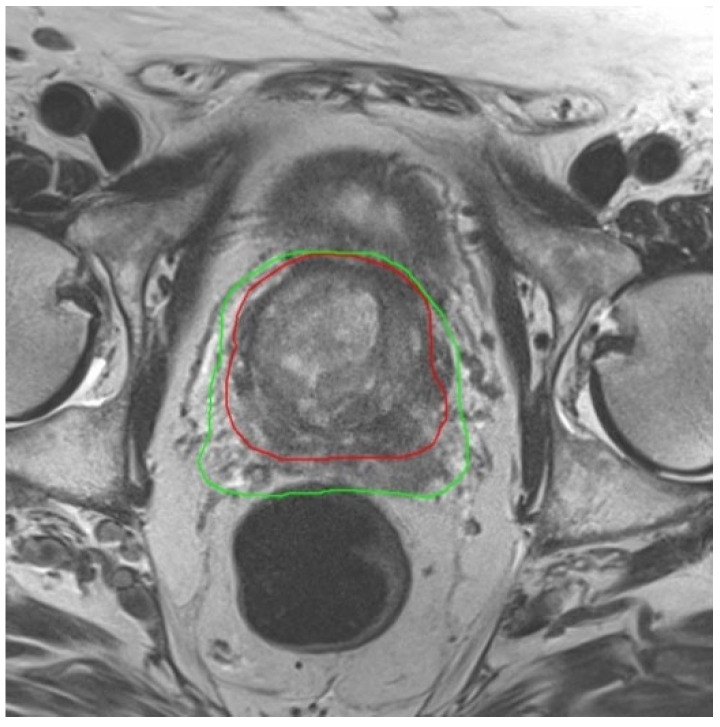
Image of MRI 1 acquired before beginning of ADT with the 60 Gy Isodose-line (green) in treatment plan. The red line is the 60 Gy Isodose-line from treatment plan based on MRI 2 after shrinkage of the prostate by ADT. The dose burden of the rectum is clearly lowered by using a new MRI after ADT.

**Figure 3:**
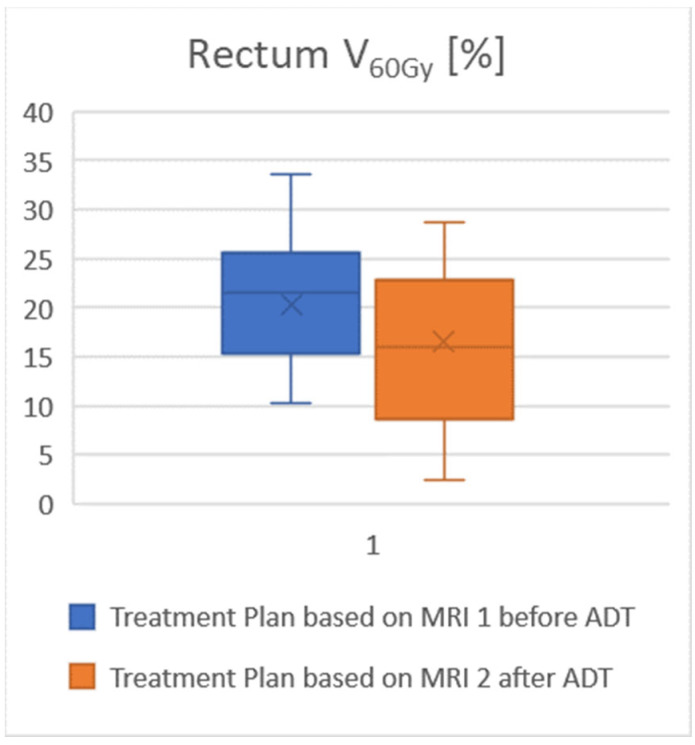
Attention to the reduction in prostate size after ADT by the updated MRI results in significant dose relief of the rectum. The proportion of the rectum exposed to more than 60 Gy is lower when treatment planning is performed using the current MRI 2.

The rectal volume averaged 76 cm^3^ (range 44–161 cm^3^). For the rectum, V_60Gy_ was 15% lower in the plans based on MRI 2 compared to a plan using MRI 1, and V_65Gy_ was as much as 23% lower. For V_60Gy_ and V_65Gy_ the relief of the rectum provided by using MRI 2 was significant. In this context, Figure 2 illustrates the course of the 60 Gy-Isodoseline. The volume of the urinary bladder averaged 192 cm^3^ (range 57–456 cm^3^). For the urinary bladder, V_50Gy_ was 10% lower in the plans based on MRI 2 compared to using MRI 1, V_65Gy_ was 14% lower, and V_70Gy_ was 21% lower. The mean dose of both risk organs in MRI 2 was approximately 1 Gy lower than the mean dose from MRI 1. In addition, for comparative plans using only the planning CT scan without any MRI, the doses to the risk organs were very similar to those in MRI 1. An overview of the exposure of organs at risk is given in Table 4; see also the visualization in Figure 3.

## 4. Discussion

To our knowledge, we are the first to quantitatively analyze whether it is worthwhile to acquire a further MRI after ADT to optimize radiotherapy treatment planning for prostate cancer. A planning CT scan alone–although acquired after ADT–systematically overestimated the volume of the prostate due to the surrounding venous plexus so that the dose to the organs at risk in the CT-based plans alone was comparable to that in the plans using MRI 1 (acquired before ADT). Because of the higher boost dose for the over estimated prostate, especially in the high-dose range, the exposure of organs at risk is significantly greater after CT-only planning. The size of the seminal vesicles is less likely to be overestimated on CT because the seminal vesicles can be clearly delineated against the surrounding adipose tissue, even on CT. For the treatment planning of the PTV prostate and seminal vesicles, a waiver of the MRI would not yet be serious. However, in the planning of the boost for the prostate, a waiver of the MRI would be particularly serious. This detail is well visible in Table 4: particularly in the high-dose range, the relief of the rectum by using MRI 2 becomes obvious. Especially here, the differences are also statistically significant, while in the low-dose area, the relief is neither statistically nor clinically relevant. Preferential sparing of the rectum over the urinary bladder in the Pareto optimization of the planning software resulted in significant sparing of the rectum in the MRI 2-based plan compared to the MRI 1-based plan, whereas sparing of the urinary bladder just missed significance. Although our study was conducted in only 17 patients, there was a significant difference in treatment planning. In a further study, it must be observed whether the reduced exposure of the rectum also translates into clinically lower GI toxicity.

However, further reduction in the CTV brings the risk of missing microscopic tumor seeding outside the prostate capsule. These reduced CTVs require image-guided radio therapy (IGRT). In addition, their safety in oncological outcome needs to be evaluated in further studies. In addition to the use of adaptive MRI-based planning after ADT, the use of a spacer interposed between prostate and rectum may also reduce the exposure of the rectum but was not in use in this trial.

A CT at the time of primary diagnosis before the initiation of ADT was not acquired in this study, and treatment plans based on such an early CT would likely have resulted in even higher burdens on the organs at risk. The principal advantage of MRI-based radiotherapy treatment planning in prostate cancer has been known in the literature for years [22,23,24,25,26]. The reduction in prostate size under ADT measured in this study was within the range of other published studies [27,28,29]. The timing of starting ADT before or concurrently with radiotherapy is controversial for oncological efficacy [30,31]. However, waiting for the prostate to shrink after a few months of ADT contributes to relief of the organs at risk and to lower toxicity. Without performing a second MRI after the effect of ADT, the volume reduction in the prostate could not be detected and the sparing of the risk organs achieved by the reduction could not be realized. The low interobserver variability known from the literature [22,32] when using the combination of CT and MRI was also reproduced in the present study with very low deviation.

It is known from several studies [33,34,35,36] that even a small decrease in mean dose and especially relief in the high dose range is associated with a significantly lower rate of proctitis and cystitis, even if these data were not yet clearly reproducible in the patient reported outcome [37]. The clinical relevance of the present results is thus demonstrated.

## 5. Conclusions

The acquisition of a second MRI after the effect of ADT can thus be recommended for MRI-based radiation planning, even if the availability of an MRI is not universal [38]. According to the results of our study, the use of an outdated MRI for radiotherapy planning should no longer be tolerated, not even if the acquisition of a second MRI is costly in the organization and delays the start of radiotherapy. However, the use of reduced CTVs requires application as IGRT, and the oncological outcome needs to be verified in further studies.

## Figures and Tables

**Table 1:** Patient Characteristics.

	N	Mean	Standard Deviation	Minimum	Maximum
Age [years]	17	79.3	5.4	63	85
PSA Level [ng/mL]	17	26.8	24.4	6.1	95.9
Gleason Score [12]	17	7.8	1.0	6	9
Duration of ADT Before MRI 2 [weeks]	17	17.2	11.4	3.1	50.6
Rectum Volume [cm^3^]	17	76.3	30.5	43.7	161
Urinary Bladder Volume [cm^3^]	17	192.1	111.7	56.6	456.2
	**N**	**%**			
cT Stage [13]					
cT1	10	58.8			
cT2	6	35.3			
cT3	1	5.9			
cT4	0	0			
cN Stage					
cN0	15	88.2			
cN1	2	11.8			
Perineural Infiltration in Needle Biopsy					
Pn0	14	82.4			
Pn1	3	17.7			

**Table 2:** Volume of Prostate [cm^3^].

	Mean ± SD	Minimum	Maximum
Prostate Volume Before ADT [cm^3^]	88.3 ± 42.9 *	47.6	226.4
Prostate Volume After ADT [cm^3^]	66.4 ± 34.3 *	37.5	182.2
Reduction in Prostate Volume During ADT [cm^3^]	22.0 ± 15.9	3.4	57.3
Reduction in Prostate Volume During ADT [%]	23.7 ± 13.5	4.7	47.2

A *t* test for dependent samples with a two-sided α of 0.05 was performed. * Indicates *p* value < 0.05.

**Table 3:** Size of PTV [cm^3^].

Plan Based On	MRI 1	MRI 2	CT
Prostate and Seminal Vesicles	294.6 ± 78.1 *^a/^*^b^	254.9 ± 81.7 *^a^	252.7 ± 83.2 *^b^
Boost Prostate	147.6 ± 52.5 *^a^	126.4 ± 55.3 *^a/^*^b^	141.5 ± 61.1 *^b^

Repeated measures ANOVA with a two-sided α of 0.05 was applied to compare the different plans for each organ. If statistically significant, pairwise *t* tests for dependent samples were subsequently performed. (two-sided α = 0.05). *^a/^*^b^ indicate respective *p* values < 0.05 for the pairwise *t* tests, comparing *a to *a and *b to *b.

**Table 4:** Exposure of Organs at Risk.

	Exposure Level	MRI 1	MRI 2	CT
Rectum	D_mean_ [Gy]	41.2 ± 4.6	40.4 ± 5.4 *	42.4 ± 5.0 *
	V_50Gy_ [%]	35.5 ± 10.2	32.2 ± 12.9	36.7 ± 10.2
	V_60Gy_ [%]	20.3 ± 6.7 *	16.6 ± 7.9 */**	20.0 ± 5.8 **
	V_65Gy_ [%]	13.1 ± 5.0 *	10.2 ± 5.0 */**	13.0 ± 4.0 **
	V_70Gy_ [%]	5.9 ± 3.3	4.2 ± 2.4	5.8 ± 2.0
	V_75Gy_ [%]	0.1 ± 0.1 *	0.0 ± 0.0 */**	0.0 ± 0.0 **
Urinary Bladder	D_mean_ [Gy]	41.4 ± 11.9	39.9 ± 10.1	40.3 ± 11.7
	V_50Gy_ [%]	39.6 ± 16.7	35.7 ± 11.9	37.1 ± 14.0
	V_65Gy_ [%]	21.1 ± 11.4	17.8 ± 7.7	20.6 ± 8.6
	V_70Gy_ [%]	13.5 ± 8.3	10.7 ± 6.1	14.2 ± 6.5
	V_75Gy_ [%]	1.4 ± 2.0	0.9 ± 1.2	0.9 ± 0.7

Repeated measures ANOVA with a two-sided α of 0.05 was applied to compare the different plans for each exposure level. If statistically significant, pairwise *t* tests for dependent samples were subsequently performed (two-sided α = 0.05). */** indicate the respective *p* values < 0.05 for the pairwise *t* tests, comparing * to * and ** to **.

## Data Availability

Research data are stored in an institutional repository and will be shared upon request to the corresponding author.
